# Cost-Effectiveness of Genotypic Antiretroviral Resistance Testing in HIV-Infected Patients with Treatment Failure

**DOI:** 10.1371/journal.pone.0000173

**Published:** 2007-01-24

**Authors:** Pedram Sendi, Huldrych F. Günthard, Mathew Simcock, Bruno Ledergerber, Jörg Schüpbach, Manuel Battegay, for the Swiss HIV Cohort Study

**Affiliations:** 1 Division of Infectious Diseases and Hospital Epidemiology, Basel University Hospital, Basel, Switzerland; 2 Basel Institute for Clinical Epidemiology, Basel University Hospital, Basel, Switzerland; 3 Division of Infectious Diseases and Hospital Epidemiology, Zurich University Hospital, Zurich, Switzerland; 4 Swiss National Center for Retroviruses, University of Zurich, Zurich, Switzerland; Cornell University, United States of America

## Abstract

**Background:**

Genotypic antiretroviral resistance testing (GRT) in HIV infection with drug resistant virus is recommended to optimize antiretroviral therapy, in particular in patients with virological failure. We estimated the clinical effect, cost and cost-effectiveness of using GRT as compared to expert opinion in patients with antiretroviral treatment failure.

**Methods:**

We developed a mathematical model of HIV disease to describe disease progression in HIV-infected patients with treatment failure and compared the incremental impact of GRT versus expert opinion to guide antiretroviral therapy. The analysis was conducted from the health care (discount rate 4%) and societal (discount rate 2%) perspective. Outcome measures included life-expectancy, quality-adjusted life-expectancy, health care costs, productivity costs and cost-effectiveness in US Dollars per quality-adjusted life-year (QALY) gained. Clinical and economic data were extracted from the large Swiss HIV Cohort Study and clinical trials.

**Results:**

Patients whose treatment was optimized with GRT versus expert opinion had an increase in discounted life-expectancy and quality-adjusted life-expectancy of three and two weeks, respectively. Health care costs with and without GRT were $US 421,000 and $US 419,000, leading to an incremental cost-effectiveness ratio of $US 35,000 per QALY gained. In the analysis from the societal perspective, GRT versus expert opinion led to an increase in discounted life-expectancy and quality-adjusted life-expectancy of three and four weeks, respectively. Health care costs with and without GRT were $US 551,000 and $US 549,000, respectively. When productivity changes were included in the analysis, GRT was cost-saving.

**Conclusions:**

GRT for treatment optimization in HIV-infected patients with treatment failure is a cost-effective use of scarce health care resources and beneficial to the society at large.

## Introduction

The advent of potent antiretroviral therapies (ART) a decade ago has led to a substantial decline of morbidity and mortality in HIV infected patients [1]–[3]. Since then many new compounds and drug classes for the treatment of HIV infection have been developed that have substantially increased the complexity of HIV patient care [4]. The emergence of resistant mutations to antiretroviral drug compounds and classes, in addition, may jeopardize the success of HIV treatment and accelerate disease progression [4]–[6]. Genotypic antiretroviral resistance testing (GRT) helps to distinguish between antiretroviral drugs to which HIV has become resistant and compounds that effectively suppress viral replication [7]–[9].

It has been documented in randomized controlled trials that GRT based treatment optimization leads to a higher viral load reduction than standard care alone in treatment-experienced patients [8], [10], [11]. Published clinical guidelines now recommend GRT in patients with antiretroviral treatment failure or in recently infected patients who may have acquired drug-resistant virus [12]–[14]. The cost of GRT guided therapy, however, is substantial (e.g., $US 625 per test in Switzerland and $US 400–500 in the USA) [15]–[17], which has prompted a debate about the appropriate use and financing of antiretroviral resistance testing in Europe and the USA [15], [17]. Health insurance companies may still be reluctant to finance GRT although it's cost-effectiveness has been documented in a few countries.

Early and more recent cost-effectiveness studies suggested that antiretroviral treatment optimization using GRT is cost-effective [17]–[19]. However, these studies did not specifically investigate job productivity changes, did not include the results of more recent long-term studies of antiretroviral resistance testing, and did not make use of the same large homogenous database on HIV disease to describe both clinical and economic outcomes. We developed a comprehensive model of HIV disease using the Swiss HIV Cohort Study (SHCS) database to describe disease progression [20]. We included resistance testing data from long-term studies and several clinical trials, and used data from patients enrolled in the SHCS to estimate the clinical effect, cost and cost-effectiveness of treatment optimization using GRT as compared to expert opinion in patients with antiretroviral treatment failure.

## Methods

### Study design

We developed a mathematical model of HIV disease to assess the incremental impact of antiretroviral therapy guided by genotypic antiretroviral resistance testing versus expert opinion alone in patients presenting with treatment failure defined as i) a viral log reduction of <1 log HIV RNA copies/ml during the first six months after initiation of antiretroviral therapy (ART), ii) a viral load increase of >1 log HIV RNA copies/ml within two months of ART, or iii) two consecutive viral load assessments >200 copies/ml after reaching undetectable plasma HIV RNA levels (<50 copies/ml) [21]. Outcome measures included life-years, quality-adjusted life-years (QALYs), health care costs and productivity costs over a patient's lifetime. Model parameters were mainly derived from patients enrolled in the Swiss HIV Cohort Study (SHCS), one of the largest cohort studies on HIV disease with over 14'000 patients enrolled to date [20].The analyses were conducted from the health care as well as societal perspective and costs and effects were discounted at an annual rate of 4% and 2%, respectively. The discount rate from the societal perspective reflects the current interest rate in Swiss government bonds whereas the discount rate from the health care perspective reflects the rate typically used by Swiss social health insurers [22], [23].

### Disease model

A state transition model of HIV disease with mutually exclusive health states was developed to describe the course of HIV disease after antiretroviral treatment failure, the starting state of patients entering the model [24], [25]. The failing treatment schedule is then either maintained or replaced by another regimen, based on expert opinion alone or with information available from genotypic antiretroviral resistance testing. Reasons for maintaining the failing treatment regimen may include the lack of more effective treatment options, patients refusing the required number of pills per day, or stable CD4 cell count despite virological failure [21]. Patients may reach virological suppression (<50 copies/ml) or have a detectable viral load during the subsequent two years with the likelihood of virological suppression being modeled as a function of the level of resistance of the HIV to the prescribed drug regimen [21]. To model the long-term impact of treatment optimization following genotypic antiretroviral resistance testing health states in patients without AIDS in the third year and thereafter were further stratified according to CD4 cell count (strata: 0–200 cells/mm^3^, 201–500 cells/mm^3^, >500 cells/mm^3^) and viral load (<1000 copies/ml, ≥1000 copies/ml), and in patients who experienced an AIDS-indicator disease by CD4 cell count only (strata: 0–200 cells/mm^3^, 201–500 cells/mm^3^, >500 cells/mm^3^). Patients are always at risk of dying due to an HIV-related or unrelated cause and were modeled until death. The disease model was populated with data extracted from the SHCS; the starting age of patients is 33 years, the average age of patients enrolled in the SHCS, and 80% of patients are male [20], [21].

### Antiretroviral resistance testing

The probability of maintaining the failing antiretroviral regimen or switching to a new regimen with or without information available from GRT was derived from Haupts et al. [21], a study within the SHCS that assessed the impact of GRT on the selection of salvage regimens in patients presenting with treatment failure (Table 1). The prescribed drug regimen may then either contain no drugs to which a resistant mutation is reported or may contain one or more drugs to which the virus is resistant (Table 1). The probability of achieving viral suppression was then conditioned on the level of viral resistance of the virus to the final chosen drug regimen as defined in Haupts et al. [21]. Patients were repeatedly tested for the presence of HIV drug resistance mutations during the two-year follow up period, if necessary, to improve the likelihood of viral suppression [21]; patient management, adherence and virological outcomes therefore reflect a real-world setting. The distribution of patients across CD4 cell counts and viral load at the end of the two-year follow-up period was used to populate the model for projecting long-term costs and clinical outcomes over a patient's lifetime. In this subsequent model, the relative risk of experiencing treatment failure when expert opinion alone was used versus GRT in virologically suppressed patients was derived from published randomized controlled trials [8], [10], [11], [26], [27].

**Table 1:**
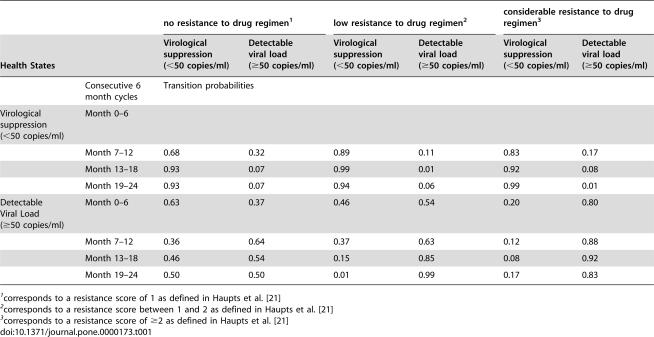
Transition probability matrix for achieving viral suppression, stratified by resistance score of the drug regimen

		no resistance to drug regimen^1^		low resistance to drug regimen^2^		considerable resistance to drug regimen^3^	
Health States		Virological suppression (<50 copies/ml)	Detectable viral load (≥50 copies/ml)	Virological suppression (<50 copies/ml)	Detectable viral load (≥50 copies/ml)	Virological suppression (<50 copies/ml)	Detectable viral load (≥50 copies/ml)
	Consecutive 6 month cycles	Transition probabilities					
Virological suppression (<50 copies/ml)	Month 0–6						
	Month 7–12	0.68	0.32	0.89	0.11	0.83	0.17
	Month 13–18	0.93	0.07	0.99	0.01	0.92	0.08
	Month 19–24	0.93	0.07	0.94	0.06	0.99	0.01
Detectable Viral Load (≥50 copies/ml)	Month 0–6	0.63	0.37	0.46	0.54	0.20	0.80
	Month 7–12	0.36	0.64	0.37	0.63	0.12	0.88
	Month 13–18	0.46	0.54	0.15	0.85	0.08	0.92
	Month 19–24	0.50	0.50	0.01	0.99	0.17	0.83

1 corresponds to a resistance score of 1 as defined in Haupts et al. [21]

2 corresponds to a resistance score between 1 and 2 as defined in Haupts et al. [21]

3 corresponds to a resistance score of ≥2 as defined in Haupts et al. [21]

### HIV disease progression

HIV disease progression was modeled by means of a transition probability matrix derived from the SHCS using the dataset from the period 1996–2004 before GRT became widely available. The probability of transitions to a health state with a viral load above or below 1000 copies/ml, a different CD4 cell stratum, the risk of developing an AIDS-indicator disease and dying was extracted from the SHCS database by pooling observations from patients on highly active antiretroviral therapy over consecutive six-month periods [28]–[30], which reflects the average period between patient visits in the SHCS (Table S1). The risk of dying from causes not related to HIV disease was derived from Swiss life tables (www.statistik.admin.ch).

### Quality of life

HIV disease may not only affect survival but may also substantially affect the patient's wellbeing [31]. In order to calculate quality-adjusted life-years (QALYs) the length of time a patient spent in a specific health state was adjusted for the quality (i.e., utility) of that state on a scale ranging from zero (death) to one (best possible health state). The utility values used in our study are derived from a study on quality of life in patients enrolled in SHCS [32] by transforming health state values assessed on a visual analogue scale into standard gamble utilities using methods as described in detail elsewhere [33]. We used regression analysis methods to derive the utilities for the different health states shown in Table 2. The disutility of experiencing an AIDS-indicator disease was derived from a meta-analysis of utility estimates in HIV-infected patients (Table 2) [31]. Since a proportion of patients may partially include the effects of ill-health on income in health state valuation [34]–[36], utilities were adjusted by increasing their value by 4.5%, as derived from Sendi and Brouwer [35]. This approach was chosen to exclude any income effects of HIV disease in QALY estimation since productivity costs are already included in monetary terms (Table 2).

**Table 2:**
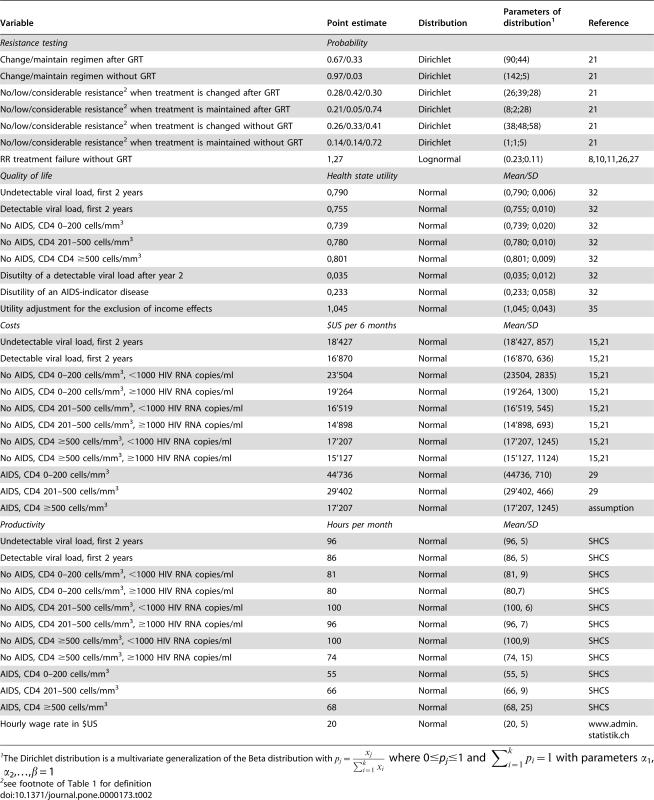
Main input variables of the cost-effectiveness model

Variable	Point estimate	Distribution	Parameters of distribution^1^	Reference
*Resistance testing*	*Probability*			
Change/maintain regimen after GRT	0.67/0.33	Dirichlet	(90;44)	21
Change/maintain regimen without GRT	0.97/0.03	Dirichlet	(142;5)	21
No/low/considerable resistance^2^ when treatment is changed after GRT	0.28/0.42/0.30	Dirichlet	(26;39;28)	21
No/low/considerable resistance^2^ when treatment is maintained after GRT	0.21/0.05/0.74	Dirichlet	(8;2;28)	21
No/low/considerable resistance^2^ when treatment is changed without GRT	0.26/0.33/0.41	Dirichlet	(38;48;58)	21
No/low/considerable resistance^2 ^when treatment is maintained without GRT	0.14/0.14/0.72	Dirichlet	(1;1;5)	21
RR treatment failure without GRT	1,27	Lognormal	(0.23;0.11)	8,10,11,26,27
*Quality of life*	*Health state utility*		*Mean/SD*	
Undetectable viral load, first 2 years	0,790	Normal	(0,790; 0,006)	32
Detectable viral load, first 2 years	0,755	Normal	(0,755; 0,010)	32
No AIDS, CD4 0–200 cells/mm^3^	0,739	Normal	(0,739; 0,020)	32
No AIDS, CD4 201–500 cells/mm^3^	0,780	Normal	(0,780; 0,010)	32
No AIDS, CD4 CD4 ≥500 cells/mm^3^	0,801	Normal	(0,801; 0,009)	32
Disutilty of a detectable viral load after year 2	0,035	Normal	(0,035; 0,012)	32
Disutility of an AIDS-indicator disease	0,233	Normal	(0,233; 0,058)	32
Utility adjustment for the exclusion of income effects	1,045	Normal	(1,045; 0,043)	35
*Costs*	*$US per 6 months*		*Mean/SD*	
Undetectable viral load, first 2 years	18'427	Normal	(18'427, 857)	15,21
Detectable viral load, first 2 years	16'870	Normal	(16'870, 636)	15,21
No AIDS, CD4 0–200 cells/mm^3^, <1000 HIV RNA copies/ml	23'504	Normal	(23504, 2835)	15,21
No AIDS, CD4 0–200 cells/mm^3^, ≥1000 HIV RNA copies/ml	19'264	Normal	(19'264, 1300)	15,21
No AIDS, CD4 201–500 cells/mm^3^, <1000 HIV RNA copies/ml	16'519	Normal	(16'519, 545)	15,21
No AIDS, CD4 201–500 cells/mm^3^, ≥1000 HIV RNA copies/ml	14'898	Normal	(14'898, 693)	15,21
No AIDS, CD4 ≥500 cells/mm^3^, <1000 HIV RNA copies/ml	17'207	Normal	(17'207, 1245)	15,21
No AIDS, CD4 ≥500 cells/mm^3^, ≥1000 HIV RNA copies/ml	15'127	Normal	(15'127, 1124)	15,21
AIDS, CD4 0–200 cells/mm^3^	44'736	Normal	(44736, 710)	29
AIDS, CD4 201–500 cells/mm^3^	29'402	Normal	(29'402, 466)	29
AIDS, CD4 ≥500 cells/mm^3^	17'207	Normal	(17'207, 1245)	assumption
*Productivity*	*Hours per month*		*Mean/SD*	
Undetectable viral load, first 2 years	96	Normal	(96, 5)	SHCS
Detectable viral load, first 2 years	86	Normal	(86, 5)	SHCS
No AIDS, CD4 0–200 cells/mm^3^, <1000 HIV RNA copies/ml	81	Normal	(81, 9)	SHCS
No AIDS, CD4 0–200 cells/mm^3^, ≥1000 HIV RNA copies/ml	80	Normal	(80,7)	SHCS
No AIDS, CD4 201–500 cells/mm^3^, <1000 HIV RNA copies/ml	100	Normal	(100, 6)	SHCS
No AIDS, CD4 201–500 cells/mm^3^, ≥1000 HIV RNA copies/ml	96	Normal	(96, 7)	SHCS
No AIDS, CD4 ≥500 cells/mm^3^, <1000 HIV RNA copies/ml	100	Normal	(100,9)	SHCS
No AIDS, CD4 ≥500 cells/mm^3^, ≥1000 HIV RNA copies/ml	74	Normal	(74, 15)	SHCS
AIDS, CD4 0–200 cells/mm^3^	55	Normal	(55, 5)	SHCS
AIDS, CD4 201–500 cells/mm^3^	66	Normal	(66, 9)	SHCS
AIDS, CD4 ≥500 cells/mm^3^	68	Normal	(68, 25)	SHCS
Hourly wage rate in $US	20	Normal	(20, 5)	www.admin.statistik.ch

1 The Dirichlet distribution is a multivariate generalization of the Beta distribution with 
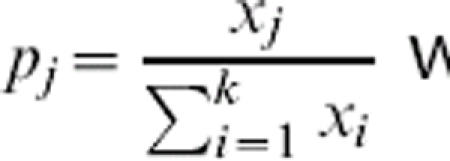
 where 0≤*p_j_*≤1 and 
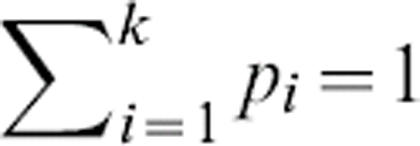
 with parameters α_1_, α_2_,…,β = 1

2 see footnote of Table 1 for definition

### Costs

In the analysis from the health care perspective we only included costs due to health care resource consumption (Table 2). We used microcosting [23] as reported in detail previously to assess the costs associated with antiretroviral therapy, drugs for the prevention of opportunistic diseases and other drugs such as antihypertensive, lipid-lowering or antidiabetic agents [15], [29]. Ambulatory costs included the costs associated with a doctor's visit, CD4 cell count and viral load measurements, blood chemistry, blood count and other diagnostic procedures such as radiological, cardiovascular and endoscopic examinations [15], [29]. The cost of GRT was $US 625 [15]. To approximate in-patient costs we used charges to Swiss health insurers for patient who stayed a minimum of one night in a hospital [15]. All costs were expressed in 2005 $US using the Swiss consumer price index for health care and the exchange rate of July 1^st^, 2005 ($US 100 = CHF 128, www.oanada.com).

With the advent of potent antiretroviral therapy HIV infection has become a chronic disease, predominantly in young patients in the working age [3]. It is therefore important to include in the analysis from the societal perspective the impact of GRT-based antiretroviral treatment optimization on the patients' ability to work and hence productivity [37]. The number of hours a patient worked was recorded during each 6-month visit where SHCS enrollees are regularly examined [29]. We therefore extracted the number of hours a patient worked in each health state as defined above from the SHCS database and attached an average Swiss wage rate of $US 20 per hour (www.statistik.admin.ch) to estimate productivity changes over a patient's simulated lifetime (Table 2). The difference in productivity between patients whose treatment were optimized with GRT versus expert opinion only was then subtracted from the health care costs associated with GRT [38], [39].

### Sensitivity analysis

We used probabilistic sensitivity analysis to assess the uncertainty around the cost and effect estimates as recommended by most recent guidelines [23], [40], [41].

Hereby a distribution is ascribed to each model input parameter using Bayesian methodology and noninfomative prior distributions [42]–[44]. We used normal distributions for cost and quality of life estimates with the corresponding standard error to model uncertainty associated with the mean input parameters, and we used a log-normal distribution for modeling the uncertainty associated with the relative risk of a treatment failure without GRT [44]. The uncertainty around count variables were modeled by means of a Dirichlet distribution, the conjugate distribution of the multinomial distribution for modeling the probability of events [45]. We used Monte Carlo simulation to estimate the uncertainty around the point estimate of incremental costs and effects of GRT versus expert opinion by sampling 5000 times from all input distributions and then recalculating the model using specialized software (TreeAge Pro 2005, TreeAge Software Inc., Williamstown, USA). The joint distribution of the resulting incremental costs and effects in our model were then summarized in terms of cost-effectiveness acceptability curves that describe the probability that the intervention is cost-effective as a function of the maximum willingness to pay per QALY gained [46], [47].

## Results

### Health care perspective

The main results of our analysis using the base case input parameters are shown in Table 3. Patients whose treatment was optimized with information available from GRT versus expert opinion had an increase in undiscounted life-expectancy and quality-adjusted life-expectancy of 2 and 6 weeks, respectively. When a 4% discount rate was used these figures were 3 and 2 weeks (Table 3). Undiscounted health care costs with and without GRT were $US 763'000 and $US 761'000, respectively, and $US 421'000 and 419'000 when a 4% discount rate was used. This corresponds to an expected cost-effectiveness ratio of $US 35'000 per QALY gained (Table 4).

**Table 3:**
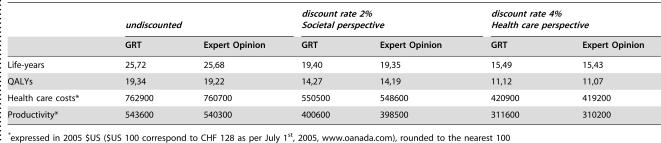
Health care costs, productivity, life-years and quality-adjusted life-years (QALY) with and without genotypic resistance testing (GRT)

	*undiscounted*		*discount rate 2% Societal perspective*		*discount rate 4% Health care perspective*	
	GRT	Expert Opinion	GRT	Expert Opinion	GRT	Expert Opinion
Life-years	25,72	25,68	19,40	19,35	15,49	15,43
QALYs	19,34	19,22	14,27	14,19	11,12	11,07
Health care costs*	762900	760700	550500	548600	420900	419200
Productivity*	543600	540300	400600	398500	311600	310200

* expressed in 2005 $US ($US 100 correspond to CHF 128 as per July 1^st^, 2005, www.oanada.com), rounded to the nearest 100

**Table 4:**
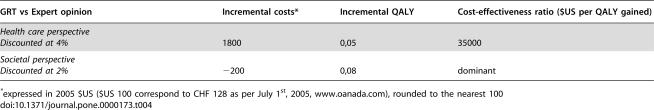
Health care costs and societal costs per quality-adjusted life-year (QALY) gained of genotypic resistance testing (GRT) versus expert opinion

GRT vs Expert opinion	Incremental costs*	Incremental QALY	Cost-effectiveness ratio ($US per QALY gained)
*Health care perspective Discounted at 4%*	1800	0,05	35000
*Societal perspective Discounted at 2%*	−200	0,08	dominant

* expressed in 2005 $US ($US 100 correspond to CHF 128 as per July 1^st^, 2005, www.oanada.com), rounded to the nearest 100

**Figure 1:**
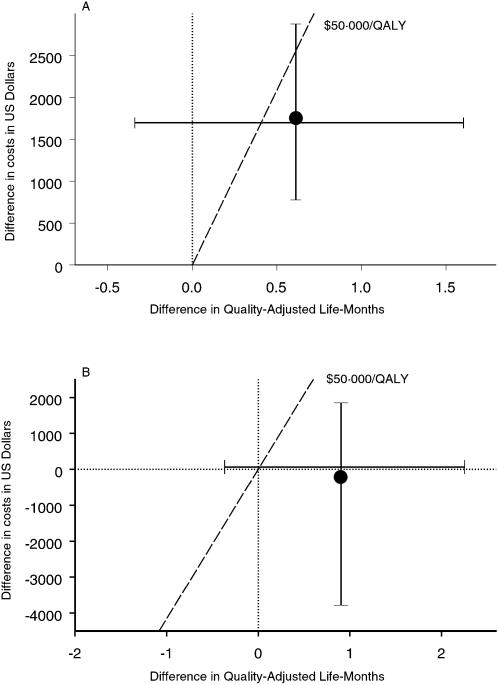
Difference in costs and effects between genotypic antiretroviral resistance testing and expert opinion for treatment optimization in HIV infected patients with treatment failure from the health care (A) and societal (B) perspective. Bars indicate the 95% credible intervals for incremental costs and effects. One quality-adjusted life-year corresponds to 12 quality-adjusted life-months. The bars cross each other at the median. The mean point estimate of the bivariate distribution of incremental costs and effects is indicated as a dot. Dotted horizontal and vertical lines at zero indicate no difference between costs and effects of the two strategies. The broken lines indicates a threshold (i.e., maximum willingness to pay) of $US 50'000 per QALY gained. The area to the right of the threshold line (i.e., the point estimate and the respective part of the distribution) is considered as cost-effective if the decision-maker's maximum willingness to pay per QALY is $US 50'000.

**Figure 2:**
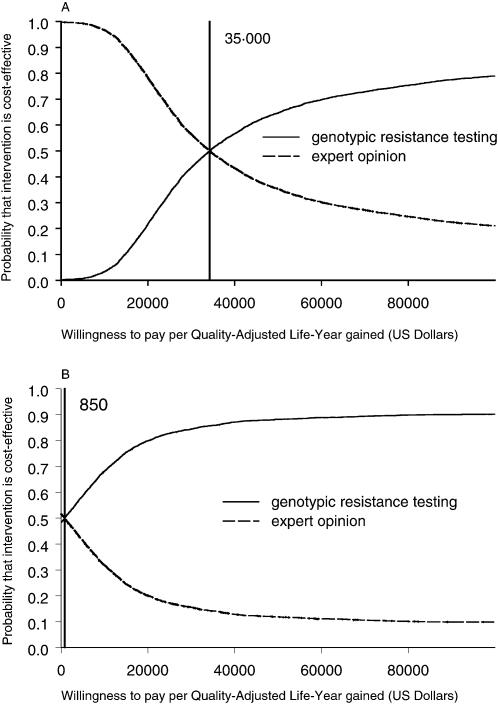
Cost-effectiveness acceptability curves from the health care (A) and societal (B) perspective. Vertical lines indicate the threshold where genotypic antiretroviral resistance testing and treatment optimization based on expert opinion have the same probability of being cost-effective. At a higher willingness to pay per QALY gained (e.g. at $US 50000/QALY) genotypic antiretroviral resistance testing has a higher probability of being cost-effective than expert opinion alone.

The point estimate and 95% credible intervals of incremental costs and effects are shown in Figure 1A. The probability that GRT will lead to a better health outcome is 89% and there is a 95% probability that incremental quality-adjusted life-months (QALMs) lie between −0.4 and 1.6. The probability that GRT will increase health care costs is 100% with the additional costs lying between $US 800 and $US 2900 with 95% probability. Summarizing the uncertainty around cost and effect estimates in terms of cost-effectiveness acceptability curves, i.e. the probability that the intervention is cost-effective for all possible willingness to pay per QALY gained, we see that at a willingness to pay of $US 35'000 or more per QALY gained GRT is the preferred treatment option (Figure 2A).

### Societal perspective

The main results of our analysis from the societal perspective, i.e. including the effects of ill-health on income in monetary terms, are shown in Table 3. Patients whose treatment was optimized with information available from GRT versus expert opinion had an increase in life-expectancy and quality-adjusted life-expectancy of 3 and 4 weeks when a 2% discount rate was used (Table 3). Discounted health care costs with and without GRT were $US 551'000 and $US 549'000, respectively (Table 3). Patients who did not receive GRT, however, had an expected discounted income of $US 399'000 versus $US 401'000 when treatment was optimized with help of GRT (Table 3). This gain in productivity more than offsets the additional health care costs due to GRT (Table 3 and 4) and GRT is therefore a dominant strategy from the societal perspective.

The point estimate and 95% credible intervals of incremental costs and effects are shown in Figure 1B. The probability that GRT will lead to a better health outcome is 91% and there is a 95% probability that incremental QALMs lie between −0.4 and 2.3. The probability that GRT will increase health care costs is 52% with the incremental costs lying between $US −3800 and $US 1900 with 95% probability. Since the societal costs including productivity changes are skewed towards negative values, the point estimate of incremental costs leads to cost savings (Table 3) whereas the probability of achieving cost savings with GRT compared to expert opinion is slightly lower than 50%. Summarizing the uncertainty around cost and effect estimates in terms of cost-effectiveness acceptability curves, i.e. the probability that the intervention is cost-effective for all possible willingness to pay per QALY gained, we see that at a willingness to pay of $US 850 or more per QALY gained GRT is the preferred treatment option (Figure 2B). Using the widely mentioned societal threshold of $US 50'000 per QALY, GRT is cost-effective with a probability of 88%.

## Discussion

We found that GRT increases projected life-expectancy and quality-adjusted life-expectancy by 2 and 6 weeks, respectively. This increase in quality-adjusted survival is clinically meaningful and comparable to the benefit of elective surgery in patients with symptomatic gallstones (gain in life-expectancy 1.7 months) or the benefit of Hepatitis B vaccination in newborn babies whose mothers have Hepatitis B (gain in life-expectancy 2 weeks) [48]. GRT is a dominant strategy from the societal perspective as the additional health care costs incurred by adding GRT to the treatment plan are more than offset by the increase in job productivity in HIV infected patients. When we included the uncertainty with respect to the input parameters of the model in the analysis, GRT has an 88% probability of being cost-effective when we used $US 50'000 per QALY gained as a threshold to determine whether an intervention represents value for money.

However, when only health care costs are considered, the cost-effectiveness ratio of GRT is $US 35'000 per QALY gained, which is similar to the cost-effectiveness ratio of implantable cardioverter-defibrillators ($US 35'000 per QALY gained) [49] and more cost-effective than other HIV treatment efforts such as antibiotic prophylaxis against *M. avium* complex infection in patients with AIDS ($US 80'000 per QALY gained) [42] and non-HIV interventions such as the use of drug-eluting stents in patients with coronary stenosis ($US 80'000 per QALY gained) [50].

In a previous study we have shown that GRT reduces health care costs over a two-year follow-up period due to the reduction of ambulatory and in-patient costs and a reduced consumption of non-HIV medication [15]. When compared to treatment optimization based on expert opinion, GRT increases overall health care costs by $US 1800 only (Table 4). The inclusion of productivity costs in our analysis, however, has a major impact on the results because with newer antiretroviral therapy HIV infection has become a chronic disease and patients, mostly adults in their working age, retain their ability to work [29], [37]. This is particularly true in countries with a low unemployment rate such as Switzerland.

In the present paper we also considered the most recent methodological developments with respect to the inclusion of productivity costs in economic evaluation to avoid a biased cost-effectiveness ratio [34], [35]. Since current evidence suggests that respondents or patients do not consistently include the effects of ill-health on income in health state valuation, omitting productivity costs in monetary terms from the analysis may lead to an underestimation of job productivity changes [35]. On the other hand, including productivity costs without adjusting utility weights in cost-effectiveness analysis can lead to an overestimation of job productivity changes [35]. We therefore included productivity costs in monetary terms and used adjusted utility weights to avoid double-counting of productivity changes.

Our study has several limitations. We used a mathematical model to approximate the real-world and project long-term costs and outcomes of GRT. However, we used best available evidence from one of the largest cohort studies on HIV disease to reflect a real-world setting [20]. The gain in (quality-adjusted) life-expectancy is slightly more conservative than those reported by other groups, which range from 1.3 months to 1.4 years [17], [18]. These differences could be due to differences in modeling natural disease history. However, our estimate is in line with the conservative scenario of Corzillius et al. [18] and supports the modeling process validity of both research groups [51]. Furthermore, we did not explicitly model patient compliance, which has been shown to substantially influence results [52]. Patient compliance, however, is already incorporated in the estimation of transition probabilities derived from the SHCS. In addition, resistance accumulation reduces viral fitness and may maintain CD4 cell counts over prolonged periods associated with clinical non-progression for some time. We explicitly modeled the possibility of maintaining the failing antiretroviral regimen, which reduces the short-term chance of viral suppression but may prevent the development of multi-resistant virus and hence preserves future drug options [53].

Our study has been conducted in Switzerland where health insurance is compulsory by law for all patients. However, our results are equally relevant for other countries such as the USA where health insurance is often funded by employers. The inclusion of GRT in health insurance plans not only leads to a benefit to the patient but also to the employer by increasing job productivity. Public health insurance coverage in the USA, on the other hand, is tied to disability status. Patients with disability status on public health insurance may also benefit from GRT based treatment optimization, as a better health state may increase the likelihood to return to work. Our results suggest that GRT represents high value for money and should be offered to all patients who can benefit from it. 

## Supporting Information

Table S1 Transition probability matrix for HIV disease progression generated by pooling consecutive six-month observations from patients on HAART enrolled in the SHCS between 1996–2004. Numbers indicate observations, the likelihood is shown in brackets. To calculate the posterior probability with a non-informative prior, the number of observations in each cell with an allowed transition is increased by one.
(0.05 MB DOC)
