# Supplementary material for: Cost-Effectiveness of Genotypic Antiretroviral Resistance Testing in HIV-Infected Patients with Treatment Failure
Source: PLoS One. 2007 Jan 24;2(1):e173. doi: 10.1371/journal.pone.0000173 (PMC1769464; doi:10.1371/journal.pone.0000173)
Supplement: Table S1 — Transition probability matrix for HIV disease progression generated by pooling consecutive six-month observations from patients on HAART enrolled in the SHCS between 1996–2004. Numbers indicate observations, the likelihood is shown in brackets. To calculate the posterior probability with a non-informative prior, the number of observations in each cell with an allowed transition is increased by one. (0.05 MB DOC) [file pone.0000173.s001.doc]

Table S1. Transition probability matrix for HIV disease progression generated by pooling consecutive six-month observations from patients on HAART enrolled in the SHCS between 1996-2004.

| *Health State* |  |  | No AIDS | | | | | | AIDS | | | Death |
| --- | --- | --- | --- | --- | --- | --- | --- | --- | --- | --- | --- | --- |
|  | *CD4 cell stratum*  *Cells/mm3* |  | 0-200 | | 201-500 | | >500 | | 0-200 | 201-500 | >500 |  |
|  |  | *HIV RNA copies/ml* | <1000 | ≥1000 | <1000 | ≥1000 | <1000 | ≥1000 |  | | | |
| No AIDS | 0-200 | <1000 | 617 (0.5347) | 81 (0.0702) | 377 (0.3267) | 30 (0.0260) | 7 (0.0061) | 0 (0.0000) | 9 (0.0078) | 0 (0.0000) | 0 (0.0000) | 33 (0.0286) |
|  |  | ≥1000 | 206 (0.2220) | 372 (0.4009) | 178 (0.1918) | 99 (0.1067) | 6 (0.0065) | 1 (0.0011) | 27 (0.0291) | 10 (0.0108) | 0 (0.0000) | 29 (0.0313) |
|  | 201-500 | <1000 | 153 (0.0333) | 56 (0.0122) | 3118 (0.6789) | 282 (0.0614) | 890 (0.1938) | 46 (0.0100) | 4 (0.0009) | 13 (0.0028) | 1 (0.0002) | 30 (0.0065) |
|  |  | ≥1000 | 36 (0.0191) | 155 (0.0824) | 422 (0.2243) | 931 (0.4949) | 168 (0.0893) | 134 (0.0712) | 7 (0.0037) | 7 (0.0037) | 0 (0.0000) | 21 (0.0112) |
|  | >500 | <1000 | 1 (0.0002) | 2 (0.0004) | 571 (0.1214) | 154 (0.0327) | 3685 (0.7834) | 265 (0.0563) | 0 (0.0000) | 3 (0.0006) | 5 (0.0011) | 18 (0.0038) |
|  |  | ≥1000 | 0 (0.0000) | 7 (0.0080) | 28 (0.0319) | 246 (0.2805) | 194 (0.2212) | 396 (0.4515) | 0 (0.0000) | 0 (0.0000) | 1 (0.0011) | 5 (0.0057) |
| AIDS | 0-200 |  | | | | | | | 1614 (0.6819) | 587 (0.2480) | 6 (0.0025) | 160 (0.0676) |
|  | 201-500 |  |  |  |  |  |  |  | 241 (0.0838) | 2184 (0.7597) | 415 (0.1443) | 35 (0.0122) |
|  | >500 |  |  |  |  |  |  |  | 9 (0.0079) | 276 (0.2410) | 856 (0.7476) | 4 (0.0035) |
| Death |  |  |  |  |  |  |  |  |  | | | (1.0000) |

Numbers indicate observations, the likelihood is shown in brackets. To calculate the posterior probability with a non-informative prior, the number of observations in each cell with an allowed transition is increased by one.
